# Cancer risk of incremental exposure to polycyclic aromatic hydrocarbons in electrocautery smoke for mastectomy personnel

**DOI:** 10.1186/1477-7819-12-31

**Published:** 2014-02-04

**Authors:** Hsin-Shun Tseng, Shi-Ping Liu, Shi-Nian Uang, Li-Ru Yang, Shien-Chih Lee, Yao-Jen Liu, Dar-Ren Chen

**Affiliations:** 1 Comprehensive Breast Cancer Center, Changhua Christian Hospital, 135 Nanhsiao Street, Changhua 50006, Taiwan; 2 Department of Public Health, Fu-Jen Catholic University, 510 Zhongzheng Road, New Taipei 24205, Taiwan; 3 Institute of Occupational Safety and Health, Council of Labor Affairs, 99, Lane 407, Hengke Road, New Taipei 22143, Taiwan

**Keywords:** Cancer risk, Electrocautery smoke, Polycyclic aromatic hydrocarbons, Surgical staff, Toxicity equivalency factor

## Abstract

**Background:**

Electrocautery applications in surgical operations produce evasive odorous smoke in the cleanest operation rooms. Because of the incomplete combustion of electrical current in the tissues and blood vessels during electrocautery applications, electrocautery smoke (ES) containing significant unknown chemicals and biological forms is released. The potential hazards and cancer risk should be further investigated from the perspective of the occupational health of surgical staff.

**Methods:**

The particle number concentration and the concentration of polycyclic aromatic hydrocarbons (PAHs) in ES were thoroughly investigated in 10 mastectomies to estimate the cancer risk for surgical staff. The particle number concentration and gaseous/particle PAHs at the surgeons’ and anesthetic technologists’ (AT) breathing heights were measured with a particle counter and filter/adsorbent samplers. PAHs were soxhlet-extracted, cleaned, and analyzed by gas chromatography/mass spectrometry.

**Results:**

Abundant submicron particles and high PAH concentrations were found in ES during regular surgical mastectomies. Most particles in ES were in the size range of 0.3 to 0.5 μm, which may potentially penetrate through the medical masks into human respiration. The average particle/gaseous phase PAH concentrations at the surgeon’s breathing height were 131 and 1,415 ng/m^3^, respectively, which is 20 to 30 times higher than those in regular outdoor environments. By using a toxicity equivalency factor, the cancer risk for the surgeons and anesthetic technologists was calculated to be 117 × 10^-6^ and 270 × 10^-6^, respectively; the higher cancer risk for anesthetic technologists arises due to the longer working hours in operation rooms.

**Conclusions:**

The carcinogenic effects of PAHs in ES on the occupational health of surgical staff should not be neglected. The use of an effective ES evacuator or smoke removal apparatus is strongly suggested to diminish the ES hazards to surgical staff.

## Background

Although operation rooms (ORs) in medical facilities are regarded as the cleanest environments because of surgical sterility requirements, surgical smoke produced by the use of electrocautery or laser systems is inevitable in modern surgery and potentially harmful to surgical personnel with long-term exposure [1]. In open surgery, the heating process of electrocautery or laser applications for tissue dissection and vessel coagulation generates a noticeable noxious plume with an undesirable odor, and gaseous and aerosol emissions from the patient’s surgical field. Electrocautery smoke (ES) contains various carcinogens and may pose unknown cancer risks for the nearly one million surgical staff around the world [2].

Airborne particles have consistently been associated with adverse cardiovascular health outcomes [3]. In ORs, surgical smoke, consisting of chemical gases and particles with various sizes, raises potential health concerns to surgical personnel in the long-term medical practice. In surgical smoke, contaminants such as hydrocarbons, phenols, nitriles, fatty acids, acrylonitrile, and carbon monoxide [4,5] and viable cellular elements [6-8] pose potential acute or long-term health hazards to surgical staff. The ES released during reduction mammoplasty was found to be mutagenic to the TA98 strain by the Ames tests [9]. Regardless of the apparent cleanliness and sterility of ORs, viral infection and the long-term health effects of chemicals in surgical smoke have drawn increasing attention from surgical personnel [10] as well as patients that have undergone surgery [11]. Smoke evacuation in ORs has been strongly suggested to minimize exposure and related effects on health [1,12].

In the OR, surgical smoke produced by electrocautery or laser applications during surgery emits tremendous amounts of particles and gases into the surrounding air. The contaminated air first passes through the OR corner vents, and is then filtered by the high efficiency particulate air filters (HEPA) and re-circulated in the OR. HEPA efficiency and the air exchange rate determine OR air quality. In general, particle number concentration is a good indicator to reveal the air quality and sterility in different ORs. However, the polluting gases in the OR should not be neglected because HEPA can only effectively remove airborne particles.

Polycyclic aromatic hydrocarbons (PAHs) are ubiquitous environmental pollutants and are the main byproducts of incomplete combustion. The exposure and the related carcinogenic effects of PAHs have been widely investigated [13,14]. Applications of high-frequency electrocautery in cutting and coagulation modes produce odorous smoke from tissue pyrolysis, which may contain significant amounts of PAHs. PAHs are distributed between gaseous and particle phases based on electrocautery power, temperature, particle concentration, molecular weight, etc. Both gaseous and particle-bound PAHs enter the human lung to a certain extent during respiration, and further induce a potential health threat. The US Environmental Protection Agency has listed 16 typical PAHs [15] as priority chemicals because of their toxicity and carcinogenic effects [16].

Among these PAHs, benzo[a]pyrene (BaP) is the most significant carcinogenic compound [17] and is often used as an indicator of cancer risk for target populations. Toxicity equivalency factors (TEFs) were recommended for calculating the relative toxicity of individual PAHs to BaP for the purpose of simplifying risk assessment [18]. This paper investigated particle number concentrations, size distribution, and gaseous and particle phase PAHs as the tracers of surgical smoke in ORs. By investigating PAH concentrations for different surgical personnel and BaP unit risk, the potential cancer risk for surgical staff exposed to ES can be estimated.

## Methods

### Sampling

ORs with similar dimensions and air exchange rates at two different hospitals were selected as the sampling environments. In order to collect detectable ES samples, mastectomy was selected as the target operation because of the longer operation time and noteworthy electrocautery usage. Since the clean room classification is a general guideline of environmental cleanliness and sterility for various ORs in hospitals, ORs with a clean room level of 10^4^ (particle number concentration with particle size greater than 0.5 μm should be less than 10^4^/ft^3^) were chosen for mastectomy investigation in this study.

For sampling consistency and further comparison between different mastectomies, the electrocautery power was kept at 25 W under the coagulation mode throughout the entire study. Fourteen mastectomies were investigated by collecting ES gas/particle samples. According to the results of 2-hour pretest mastectomy, electrocautery usage time was scrupulously recorded with a laser portable particle counter (Met One, Grants Pass, Oregon, USA) monitoring particle number concentrations of particle 50% cut-sizes from 0.3, 0.5, 1, 3, 5, and 10 μm at an interval of 5 minutes until the end of surgery.

For the purpose of investigating surgical staff exposure to ES, air samples were taken within 30 cm at the breathing height of the surgeons and the anesthetic technologist (AT) without contacting the sterile region. Air samples, including particle and gaseous phases, were collected at the sampling flow rate of 30 L/min. The ES air was first passed through a 47-mm quartz fiber filter to collect smoke aerosols, and then polluted gases were trapped by the polyurethane foam of PUF-XAD-PUF adsorbent. Before surgical operation, background air samples were also collected for comparison. This study was approved by the Institutional Review Board and Ethics Committee in our hospital. Informed consent was obtained from all patients.

### PAH analysis

The PAH chemical analysis protocol included soxhlet and sonication extraction, solvent exchange, cleanup, nitrogen blow down, and the final gas chromatography/mass spectrometry (GC/MS) analysis. Prior to sample solvent extraction, samples for PAHs were spiked with a deuterium surrogate solution containing 10 ng of d8-naphthalene, d10-fluorene, d12-chrysene, and d12-perylene to monitor efficiency throughout the entire analytical process. A sonication method was modified to extract particle-bound PAHs from quartz fiber filter samples, while PUF-XAD-PUF adsorbent samples were soxhlet-extracted for 24 hours. This was followed by a solvent exchange to hexane, cleanup with 5% water deactivated silica gel, and nitrogen blow down to about 100 μL. Before GC/MS, a deuterium internal standard solution (10 ng) consisting of d10-phenanthrene, d10-pyrene, and d12-BaP was injected to analyze PAHs both qualitatively and quantitatively.

A Hewlett-Packard gas chromatography (GC, Model 6890) equipped with a mass selective detector (MS, Hewlett-Packard Model 5973) was used to quantify PAHs. The GC/MS was tuned weekly with 1 ng/μL decafluorotriphenylphosphine. A 30 m × 0.25 mm identity column of 0.25-μm film thickness DB-5MS phase (J&W Scientific, Folsom, CA, USA) was used to separate PAHs. A splitless injection system was used, with a 2-minute delay before purge. Helium was the carrier gas, operated at a constant linear velocity of 25 cm/second. The injector and transfer line were maintained at 290°C and 300°C, respectively. The GC temperature program was 50°C for one minute, 20°C/minute to 300°C, and maintained for 15 minutes; the sample injection volume was 1 μL. Selective ion monitoring in the GC/MS was used to quantify individual PAHs.

The PAHs analyzed in this study included naphthalene, acenaphthylene, acenaphthene, fluorine, phenanthrene, anthracene, fluoranthene, pyrene, benzo[a]anthracene, chrysene, benzo[b]fluoranthene, benzo[k]fluoranthene, BaP, indeno[c,d]pyrene, dibenzo[a,h]anthracene, and benzo[g,h,i] perylene. In addition, hetero-PAHs, including oxygen, sulfur, and nitrogen, were also detected: 2,3 benzofuran, dibenzofuran, dibenzothiophene, 7,8 benzoquinolene, and carbazole.

In order to positively identify trace level PAHs, a final sample volume of approximately 100 μL and GC/MS detection limits of approximately 3 to 25 pg of individual PAHs per injection were achieved. The significance of PAH concentration above the method detection limit was checked by evaluating field and laboratory blanks, and spiked PAH recovery. The method detection limit was determined as three times the standard deviation above the mean in the field blanks.

### Quality control

Field blanks, laboratory blanks, and spike tests were performed to ensure the sampling and analytical background consistency throughout the study. The blank levels were less than 0.85% of the total PAH concentration level in OR samples. Based on the surrogate recoveries, the average PAH recovery efficiency was 85% (52% to 114%). All PAH concentrations were corrected by the corresponding surrogate PAH recovery efficiencies and field blanks. Particle-bound and gaseous PAH concentrations were determined by dividing the individual PAH mass by the sampled air volume. SPSS 12.0 was used in statistical analysis.

## Results and discussion

### Electrocautery smoke (ES) monitoring in operating rooms (ORs)

ES was highly related to the original application; therefore, electrocautery usage frequency was the primary indicator for its pollution level. Table 1 shows electrocautery usage times for mastectomy at two hospitals, which occupied about one-third of the total operational time. The continuous electrocautery usage produced tremendous amounts of visible smoke particles and invisible gases in the OR air.

**Table 1:** Data related to surgical smoke during mastectomies

**Case no.**	**Operation time (minutes)**	**EC time usage (minutes)**	**PAH concentration for surgical personnel (ng/m**^**3**^**)**			
			**Surgeons**		**AT**	
			**Gas**	**Particle**	**Gas**	**Particle**
1	48	16.8	1,074	59	874	65
2	148	51.2	2,148	205	1,074	73
3	74	25.1	1,592	98	1,041	68
4	125	42.4	2,257	104	2,124	213
5	119	41.7	2,062	236	2,199	214
6	105	35.8	487	98	508	119
7	114	37.1	753	93	190	86
8	136	48.2	1,124	98	316	134
9	51	18.6	1,297	147	1,305	148
10	43	14.2	1,360	168	1,307	168
Mean ± standard deviation	96 ± 39	33.1 ± 13.5	1,415 ± 598	131 ± 57	1,094 ± 682	129 ± 57

**Figure 1:**
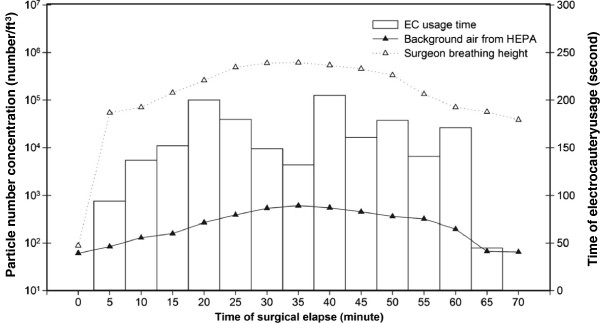
Total particle number concentration and electrocautery usage time with respect to the surgical elapse time.

To assess the pollution level of ES, particle number concentration was used as an indicator of the air quality at various OR locations, such as the clean air from the HEPA, the air under the surgical lamp where surgeons stand, at the AT position, etc. During the mastectomy, the particle number concentrations of the air under the surgical lamp reached the level of 10^6^/ft^3^ (Figure 1). Compared with the downwash clean air from the HEPA, with particle number concentrations below 10^3^/ft^3^, the enormous amount of particles and gases in ES increased excess inhalation by the surgical personnel and posed potential health hazards. With the existence of particle-bound hazardous substances, particle size becomes even more crucial in evaluating the potential damage to the human respiratory system.

**Figure 2:**
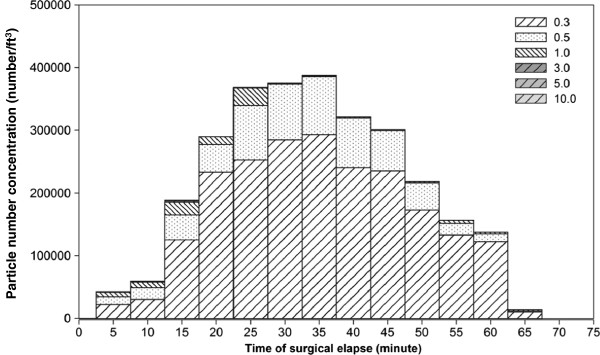
**Particle number size distribution at the surgeon’s breathing height.** Particle sizes of 0.3 and 0.5 μm dominate the total particle number concentrations.

In ES, the majority of airborne particles were 0.3 μm in size and amounted to 70% of total particles (Figure 2). Submicron particles have been regarded as respiratory particles that may penetrate deep into the bronchiole and alveoli of the lung and subsequently result in various degrees of lung diseases [19]. Particles of various sizes in the air were critical for assessing the surgical staff’s respiratory exposure. The particle number distribution at various sizes showed that the average number mean diameter of ES was 0.35 μm (95% confidence interval: 0.32 to 0.38 μm). Thus, 0.3-μm particles could be chosen as the representative particles for further ES investigation. Among the OR locations, the highest particle number concentrations (reaching 1.2 × 10^6^/ft^3^) were found in the surgeon’s breathing zone right under the surgical lamp, followed by the AT position. It is probable that the downwash air from the HEPA was hindered by the surgical lamp, and smoke recirculation was also induced by the heat from the lamp. Shifting the surgical lamp aside to enhance the clean air downwash to the stagnant ES under the surgical lamp was suggested to minimize such a polluted scenario.

### PAH levels of electrocautery smoke in the OR

**Figure 3:**
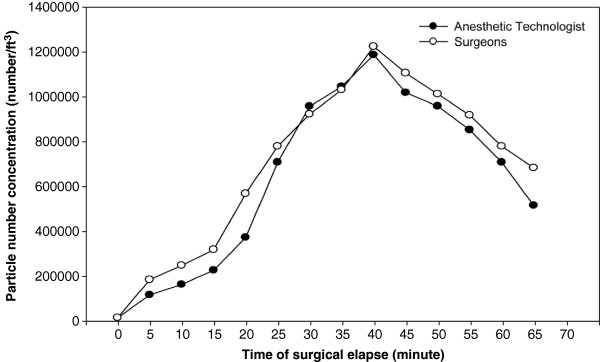
Total particle number concentrations at the breathing heights of the surgeons and ATs.

After ES enters the OR air, the downwash clean air from the ceiling HEPA dissipates the ES into the surrounding environment, resulting in ES exposure to all surgical personnel. The coincident patterns of particle number concentrations at the surgeon and AT breathing heights (Figure 3) indicated that the ES plume disperses from the surgical site to the entire OR area instantaneously. The ES inhalation health threat to other surgical staff should not be neglected. Compared with the PAH background level before surgery in the OR (PAH_Gas_ = 23.4 ± 8.6 ng/m^3^, PAH_Particle_ = 1.2 ± 0.3 ng/m^3^), the particle and gaseous PAH concentrations for the surgeons and the AT increased by nearly 40 to 100 times. The inhalation of carcinogenic PAHs in ES could further deteriorate the health of surgical personnel during mastectomy in the long-term occupational viewpoints.

In further analyses of the two hospitals involved in the study and different mastectomy operations, no statistical correlation was found between operation time, electrocautery usage time, and PAH concentrations. The air exchange rate (AER), which is controlled by the positive pressure and ventilation setups in ORs, is a critical parameter used to avoid contamination of the patient’s wounds. The AERs, determined by operational categories and necessary requirements, are difficult to measure onsite in the OR and may be the major factor in determining the PAH concentrations. A higher AER could effectively dissipate the ES pollution and decrease the particle and PAH concentrations. The consistent particle and gaseous PAH concentration results at different hospitals and in various scales of mastectomy operations revealed that the ES pollution level was statistically the same, and could be further applied in assessing the risk to surgical personnel.

**Figure 4:**
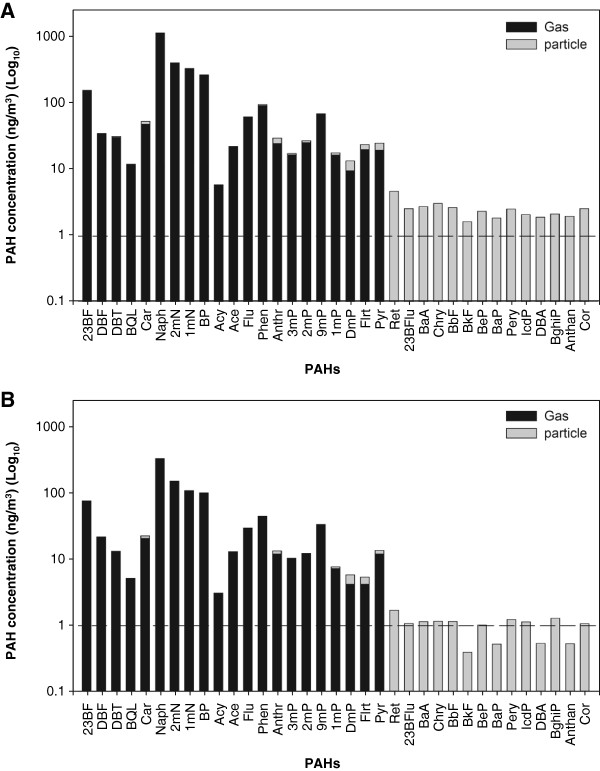
Concentration of gaseous- and particle-phase PAHs at the breathing heights of the surgeons (A) and ATs (B).

Figure 4 shows the concentrations of the particle and gaseous PAHs for the surgeon and the AT. Since the ES emission source was around the patient’s open wound during surgery and the surgeon was at the closest position, it is not surprising that the surgeon was exposed to higher levels of PAHs, roughly 1.5 times higher than that for the AT. The similarity of PAH patterns between these two major surgical personnel indicated that the ES exposure to the surgical team was from the electrocautery usage. The downwash clean air from the HEPA may not only dissipate the smoke, but also disperse ES to the surrounding area, resulting in the homogeneous PAH exposure to surgical personnel in ORs.

### Cancer risk of PAH exposure for surgical personnel

Compared with the background PAH concentrations in ORs, the ES pollution scenario substantially increases the PAH levels during operations. The surgeon’s exposure to an average of 16 typical PAHs in the gaseous and particle phases were 1,279 and 37 ng/m^3^, respectively. Though gaseous PAH concentrations were 30 times higher than those associated with particles, it should be noted that high molecular weight PAHs often result in higher carcinogenic effects [20].

BaP has been widely investigated for its mutagenic effects and is regarded as the most likely carcinogen in the PAH group. TEFs for various pollutants to a specific well-known toxic chemical are commonly used in the dose–response relationship of risk assessment [18]. Based on the possible health effects of ubiquitous PAHs, TEFs for 16 typical PAHs were applied to estimate the relative carcinogenic effects. By combining the unit risk of BaP, 8.7 × 10^-5^ (ng/m^3^)^-1^, and the corresponding gaseous and particle PAHs in the target environments, cancer risk for a 70-year lifetime exposure can be estimated. For surgical personnel, the cancer risk should be modified to unit-hour exposure risk based on their professional working time.

Table 2 lists the risk associated with gaseous and particle PAHs for both surgeons and ATs as the representatives of surgical personnel. The total lifetime cancer risks to the surgeons and ATs were 46.8 and 29.3 × 10^-6^ per hour exposure, respectively. The surgeons have a higher risk because of closer contact to the ES. Though the AT hourly exposure risk was 62% of that for the surgeons, the longer working time in the OR increased the risk considerably. Based on the unit-hour cancer risks and the associated working hours, the 70-year lifetime cancer risks for the surgeons and ATs are 117 × 10^-6^ and 270 × 10^-6^, respectively, which are significantly higher than the WHO recommended safety level of 1 × 10^-6^. Such facts indicate that the ES hazards to surgical personnel in ORs should not be neglected.

**Table 2:** **PAH and BaP**_**eq **_**concentrations for the surgeons and ATs in the OR**

**PAH**	**TEF**	**Surgeon**		**AT**	
		**PAH**_**Gas**_	**PAH**_**Particle**_	**PAH**_**Gas**_	**PAH**_**Particle**_
Naphthalene	0.001	1055	1.6	584	1.1
Acenaphthylene	0.001	5.8	ND	3.5	ND
Acenaphthene	0.001	19.9	ND	14.6	ND
Fluorene	0.001	55.4	0.8	42.2	0.5
Phenanthrene	0.001	84.3	3.2	68.3	2.2
Anthracene	0.01	22.2	5.6	16.7	4.0
Fluoranthene	0.001	18.9	3.8	14.2	2.7
Pyrene	0.001	17.8	5.4	13.3	4.1
Benzo[a]anthracene	0.1	ND	2.7	ND	1.3
Chrysene	0.01	ND	3.0	ND	1.4
Benzo[b]fluoranthene	0.1	ND	2.6	ND	1.3
Benzo[k]fluoranthene	0.1	ND	1.5	ND	0.7
BaP	1	ND	1.7	ND	1.1
Indeno[cd]pyrene	0.1	ND	1.8	ND	1.0
Dibenzo[ah]anthracene	5	ND	1.8	ND	1.1
Benzo[ghi]peryrene	0.01	ND	2.1	ND	1.5
BaP_eq_		1.5	11.4	0.9	7.2
Calculated lifetime cancer risk (×10^-6^) based on 24-hr exposure^a^		131	992	78	626
Risk of unit-hour exposure (×10^-6^)		46.8		29.3	
Average operation time (hour/day)		2.5		9.2	
Estimated lifetime cancer risk (×10^-6^)		117		270	

^a^Calculated risk based on BaP_eq_ unit risk of 8.7 × 10^-5^ (ng/m^3^)^-1^. ND, not detected.

Though the gaseous PAH concentrations outnumbered the particle-bound PAHs, the major cancer risk posed by particles (89%) shows that the ES particles should be treated with the highest priority in the removal of these health-threatening hazards. However, the cancer risk related to gaseous PAHs might be underestimated due to the contribution of the hetero-PAHs. The abundant hetero-PAHs in ES, such as 2,3-benzofuran, dibenzofuran, dibenzothiophene, 7,8-benzoquinolene, and carbazole, were also found in the gaseous phase. Hetero-PAHs are likely adapted by metabolic processes and therefore have a greater tendency to induce mutation or carcinoma. Though the HEPA can remove particles in recirculated air, gaseous pollutants may penetrate through filters and accumulate in the OR air. Effectively removing ES in ORs should be emphasized to minimize the potential adverse health effects to surgical personnel.

## Conclusions

Submicron particles in ES in the ORs during modern surgery are an inevitable nuisance and contain carcinogenic chemicals. In measuring particle number concentrations at various size ranges in mastectomy, more than 70% of ES particles were found to be smaller than 0.3 μm, indicating that the particles may threaten the health of surgical personnel through respiration. The ES exposure for surgeons and ATs has been fully investigated by measuring the PAH concentrations in both the gaseous and particle phases in their breathing zones. Based on TEFs, this study estimated that the average cancer risk in a 70-year lifetime for surgeons and ATs was estimated to be 117 × 10^-6^ and 270 × 10^-6^, respectively. Effective smoke extractors in the ORs or high-efficiency masks are suggested to minimize potential health hazards to surgical personnel.

## Abbreviations

AER: Air exchange rate; AT: Anesthetic technologist; BaP: Benzo[a]pyrene; ES: Electrocautery smoke; GC/MS: Gas chromatography/mass spectrometry; HEPA: High efficiency particulate air filter; OR: Operation rooms; PAHs: Polycyclic aromatic hydrocarbons; TEF: Toxicity equivalency factors.

## Competing interests

The authors declare that they have no competing interests.

## Authors’ contributions

HST, SPL, SNU, LRY, SCL, YJL and DRC were responsible for the study concept and design. SPL, SNU, LRY, SCL and YJL were responsible for data acquisition and conception of the manuscript. HST, SPL and SNU were responsible for statistical analysis. All authors were responsible for data analysis and interpretation. HST, SPL and DRC drafted and designed the manuscript. SPL and DRC were responsible for manuscript preparation and contributed equally to this work. All authors reviewed the manuscript. All authors read and approved the final manuscript.
